# Role of Serum Micro-RNA-122-5p Expression as a Circulatory Biomarker in People Having Both Knee Osteoarthritis and Osteoporosis: A Case-Control Study

**DOI:** 10.7759/cureus.60844

**Published:** 2024-05-22

**Authors:** Rashmi Yadav, Rajeshwar N Srivastava, Dharmendra Kumar, Amar Sharma, Sudeepti R Srivastava, Shatakshi Pant, Saloni Raj, Abbas A Mehdi, Devendra Parmar

**Affiliations:** 1 Department of Orthopedic Surgery, King George's Medical University, Lucknow, IND; 2 Department of Epidemiology and Public Health, Westminster College, Utah, USA; 3 Department of Biochemistry, King George's Medical University, Lucknow, IND; 4 Department of Developmental Toxicology, Indian Institute of Toxicology Research, Lucknow, IND

**Keywords:** kellgren and lawrence grade, expression, biomarker, mirna, osteoporosis, knee osteoarthritis

## Abstract

Background

Although knee osteoarthritis (KOA) and osteoporosis (OP) manifest distinct pathophysiologies, they share numerous similarities. These health conditions are commonly found in older individuals, particularly among women. The objective of this study is to explore the expression of micro-RNA (miRNA) 122-5p (miR-122-5p) in people affected by both KOA and OP. The main aim is to identify diagnostic biomarkers and potential therapeutic targets, which could help develop personalized treatment approaches.

Methods

As part of the study, a total of 268 serum samples were collected from the participants, who were divided into four groups: KOA, OP, KOA and OP, and controls, with 67 subjects per group. The miRNA species-containing total RNA was isolated from the serum samples using an miRNeasy serum/plasma kit by QIAGEN (Hilden, Germany). The expression of miR-122-5p was examined in each group using real-time quantitative polymerase chain reaction.

Results

Expression of miR-122-5p in all three groups (KOA, OP, and common group of KOA and OP) was significantly upregulated, and the fold change value was much higher in the group having both diseases.

Conclusions

These results might contribute to the identification of cases at risk, early diagnosis, and development, and might also contribute to the development of therapeutic targets in subjects having both KOA and OP.

## Introduction

Despite having distinct pathophysiologies, osteoarthritis (OA) and osteoporosis (OP) share notable similarities, representing prevalent musculoskeletal conditions in older individuals, predominantly affecting women. The incidence of OP and OA is rising in the ageing population, highlighting their increasing prevalence [1]. Both disorders are intricate and remain insufficiently investigated at the molecular level. Both genetic and environmental factors exert significant influence on their development. Several potential genes associated with these diseases have been identified, and, intriguingly, some of these genes are shared between OP and knee osteoarthritis (KOA), although this aspect has not been explored in individuals with both conditions [2].

OA, a persistent and incapacitating disease primarily affecting mobile joints, particularly weight-bearing synovial joints, is a leading cause of joint replacement. The overall prevalence of KOA is reported to be 28.7% [3]. OA significantly contributes to work-related impairment in individuals over 50 years of age [4] and is the most prevalent form of arthritis, especially in the knees [5]. Unfortunately, the high use and stress on this joint make it susceptible to painful conditions such as OA [6]. Given the gradual progression of the disease, timely diagnosis and treatment are crucial. Therefore, there is an urgent need for effective and reliable biomarkers for KOA. While various investigators have proposed biochemical markers based on cartilage, synovium, or bone for OA diagnosis and prognosis [7], convincing biomarkers for early detection of KOA are still limited, hindering timely intervention.

In contrast, despite genetic variations having a limited impact on gene expression and only partially explaining the disease's etiology, OP is a complex disorder with a known hereditary component [8]. It is characterized by the loss of bone mass and quality, leading to compromised bone strength [9]. Millions of fractures are reported annually, with up to 40% of postmenopausal women and 20% of males over 50 considered at risk [10]. These numbers are anticipated to rise gradually due to the ageing population, making OP a significant global health and economic challenge [11].

Several research groups [12-16] have suggested that micro-RNAs (miRNAs) could play a pivotal role in both OA and OP, as mechanical loading has been shown to influence miRNA expression. miRNAs are small non-coding RNA molecules, approximately 22 nucleotides or smaller, serving as essential molecular regulators that modify post-transcriptional gene expression by preventing the translation or initiating the degradation of specific mRNAs. Because miRNAs can target a collection of genes with similar functions and entire pathways instead of individual genes, their significance as therapeutic targets is increasing [17].

miRNAs play a significant role in controlling various biological processes, including development, cell differentiation, proliferation, death, immunity, and metabolism, as evidenced by accumulated research [18,19]. Aberrant miRNA expression has been linked to a broad spectrum of human diseases, such as cancer, viral infections, nervous system disorders, cardiovascular disorders, musculoskeletal disorders, diabetes, and others [18,20]. This suggests that using these aberrantly expressed miRNAs as biomarkers for diseases not only is a valuable diagnostic strategy but also positions these non-coding RNAs as promising candidates for new drug discovery [18,19].

Several studies have suggested that miRNA-122-5p (miR-122-5p) may play a role in the development of KOA, and its expression has been found to be altered in the synovial fluid, cartilage, and serum of individuals with KOA [21]. Similarly, miR-122-5p has been investigated in the context of OP. Research indicates that it may be involved in the dysregulation of osteoblast and osteoclast activity, bone formation, and bone remodeling processes. Studies suggest that miR-122-5p may contribute to the pathogenesis of OP by influencing bone mineral density and bone metabolism [22]. Elucidating the role of miR-122-5p as a potential shared biomarker in subjects having both diseases could have implications for diagnosis, prognosis, and therapeutic interventions targeting both conditions simultaneously. This study aims to investigate the role of miR-122-5p as a common biomarker in individuals with both KOA and OP, as its role in the comorbidity of subjects having both KOA and OP has not been fully elucidated.

## Materials and methods

Study design

A total of 268 subjects were recruited for this study, with each group comprising 67 individuals. The groups were categorized as KOA, OP, KOA and OP (common), and control. Inclusion criteria for the KOA group were as follows: 1) individuals of any gender diagnosed with OA knee according to the American College of Rheumatology (ACR) guidelines, meeting criteria such as a) knee pain with an observable osteophyte on X-ray and b) presence of at least one of the following: i) crepitus during knee range of motion, ii) age 45 years or older, iii) morning stiffness lasting less than 30 minutes; 2) confirmation of KOA through anteroposterior standing and lateral knee radiographs showing a severity equivalent to Kellgren and Lawrence (KL) grade of at least 2; and 3) subjects providing informed consent.

Exclusion criteria for the KOA group included: a) secondary OA knee, b) hypercalcemia (total serum calcium exceeding 10.5 mg/dL), c) hyperparathyroidism (parathyroid hormone exceeding 65 pg/mL), d) presence of serious medical conditions or impairments that, according to the investigator, would impede study participation, and e) intent to permanently relocate from the region during the trial period.

For the OP group, inclusion criteria comprised a) both male and female subjects, b) age over 45 years, and c) chronic back pain. Exclusion criteria for the OP group included: a) uncontrolled diabetes, b) abnormal values for blood urea creatinine, HBA1C, c) chronic renal failure or abnormal serum creatinine levels, d) THYROID disorder, e) Rheumatoid arthritis, f) gout and pseudogout, g) malignancy, h) chronic renal failure, and i) prolonged steroid intake.

It is noteworthy that the inclusion and exclusion criteria for subjects with both KOA and OP are identical to those specified for the KOA and OP groups, respectively.

Sample acquisition and handling

Around 5 mL of peripheral blood was collected. After collection, the whole blood was allowed to clot by leaving it undisturbed at room temperature for 15-30 minutes. The clot was then removed by centrifuging at 1,000-2,000 x g for 10 minutes in a refrigerated centrifuge, resulting in serum. The final supernatant was stored at -80°C.

miRNA analysis

The cell-free total RNA purification process was conducted using the miRNeasy serum/plasma kit by QIAGEN (New Delhi, India). Briefly thawed (on ice) serum samples (200μL) were subjected to lysis by adding 1mL of lysis buffer. The isolated RNA underwent cDNA synthesis using the miRCURY LNA RT kit (QIAGEN). The 5x miRCURY RT reaction buffer and 10x miRCURY RT enzyme were introduced to the isolated RNA. Subsequently, 2μL of eluent was applied to 200 μL of serum plasma, adjusting each RNA template to a concentration of 5ng/μL. The cDNA was formed from 1μL of this RNA, and 5μL of Applied Biosystems™ TaqMan reagent was added to the reaction mixture. This was followed by the addition of 0.5μL of miRNA-122-5p (miR-122-5p) specific primer and 3.5μL of nuclease-free water, resulting in a total sample volume of 10μL. The reverse transcription polymerase chain reaction (RT-PCR) procedure was executed with the following conditions: (1) hold stage (50℃ for 2 minutes and 95℃ for 10 minutes) and (2) PCR stage (95℃ for 15 seconds and 60℃ for 1 minute). ABI ThermoFisher QuantStudio 5 system (ThermoFisher Scientific India Pvt. Ltd, Mumbai, India) was used to perform RT-PCR since it provides standard quantitative PCR (qPCR) applications and functions with remote monitoring and cloud-enabled analytics with fixed 384-well blocks (Appendix).

Statistical analysis

The Statistical Package for Social Sciences (SPSS) Version 21 (ICM Corp., Armonk, NY) was used for all statistical analyses. Categorical variables were expressed as percentages (%), while continuous variables were presented as mean±SD. To determine the overall diagnostic value, a receiver operator characteristic curve (ROC) analysis was conducted. The association between the mean expression of the biomarker and the healthy control group was evaluated using a paired t-test and Mann-Whitney U test. In addition, one-way analysis of variance (ANOVA) was used for comparisons involving more than two groups, as appropriate.

## Results

miR-122-5p analysis

**Figure 1:**
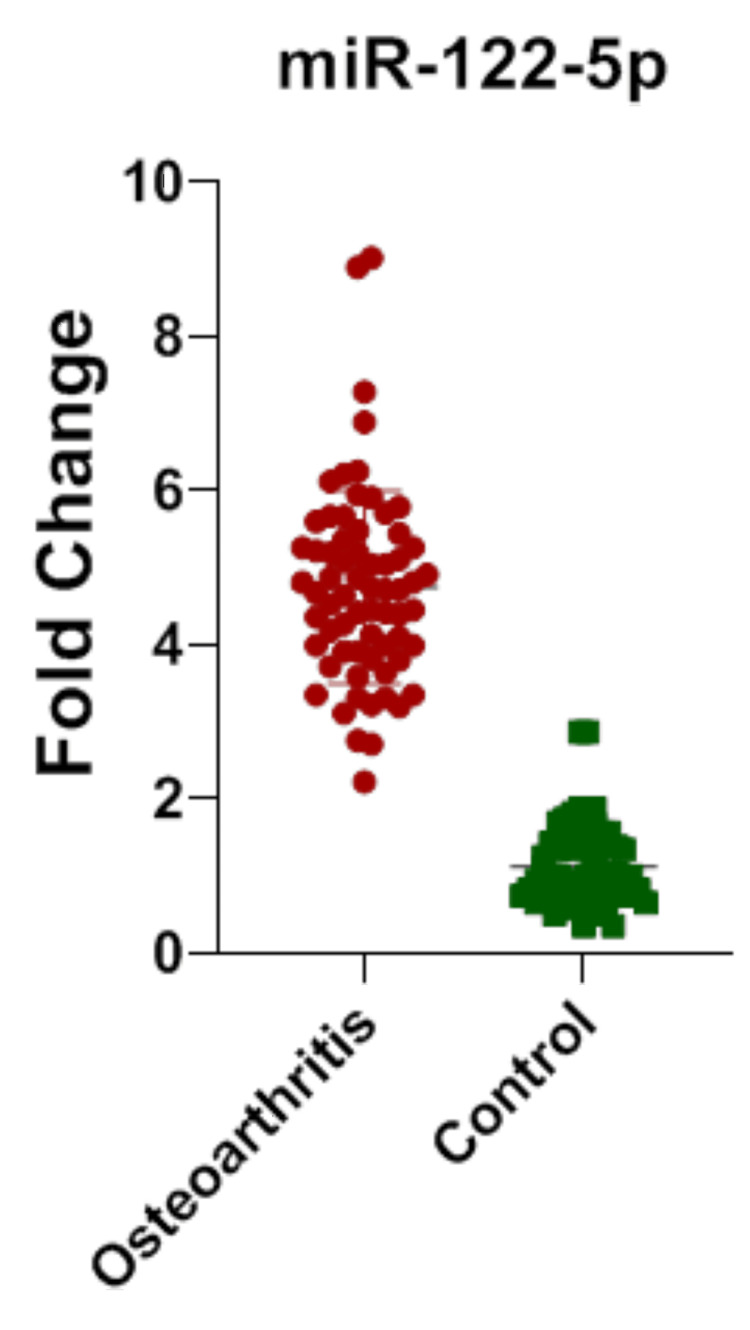
Dot plots were used to illustrate the differential distribution of miRNA levels (mean±SD) for miR-122-5p in knee osteoarthritis cases and controls. Each point on the plot indicates the individual expression level; the upper line represents the mean of the group, while the lower line represents the group's standard deviation. miR-122-5p, miRNA-122-5p

**Figure 2:**
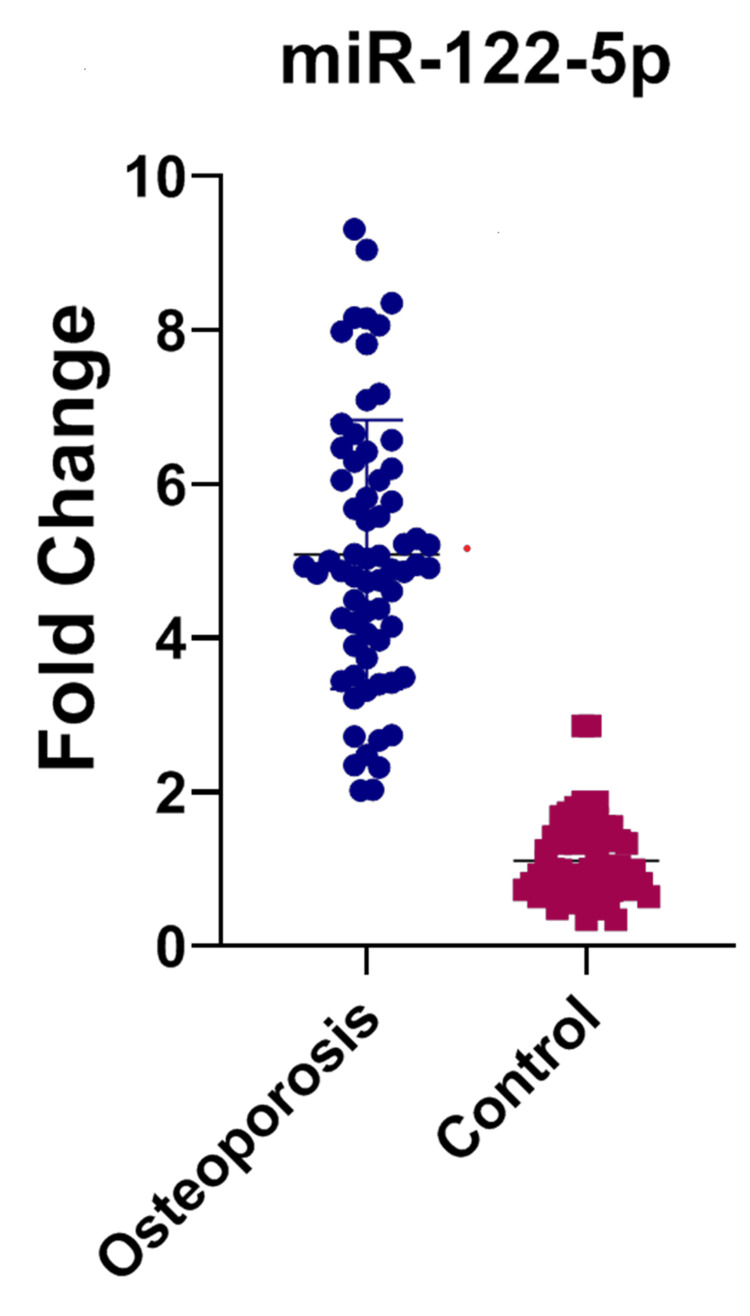
Dot plots were used to illustrate the differential distribution of miRNA levels (mean±SD) for miR-122-5p in osteoporosis cases and controls. Each point on the plot indicates the individual expression level; the upper line represents the mean of the group, while the lower line represents the group's standard deviation. miR-122-5p, miRNA-122-5p

**Figure 3:**
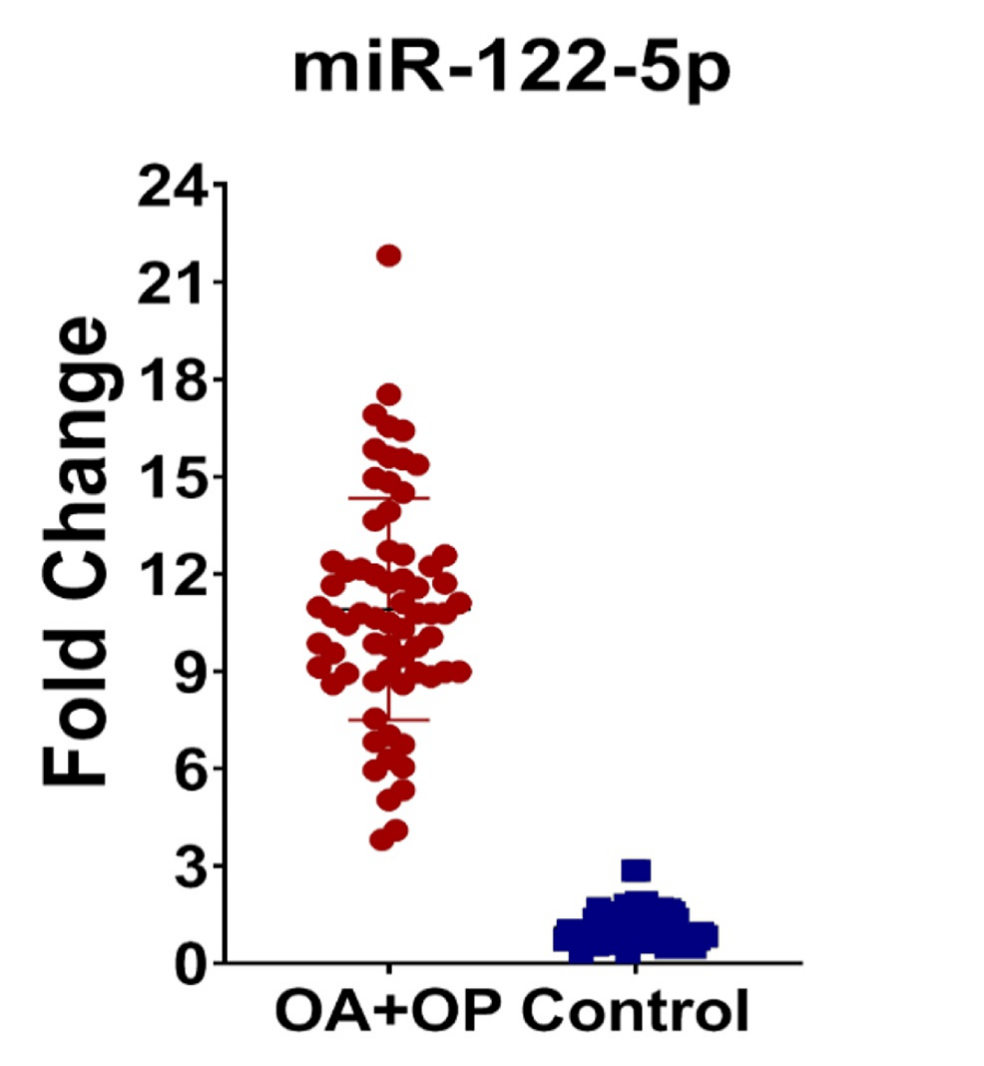
Dot plots were used to illustrate the differential distribution of miRNA levels (mean±SD) for miR-122-5p in knee OA with OP cases and controls. Each point on the plot indicates the individual expression level; the upper line represents the mean of the group, while the lower line represents the group's standard deviation. miR-122-5p, miRNA-122-5p, OA, osteoarthritis; OP, osteoporosis

**Figure 4:**
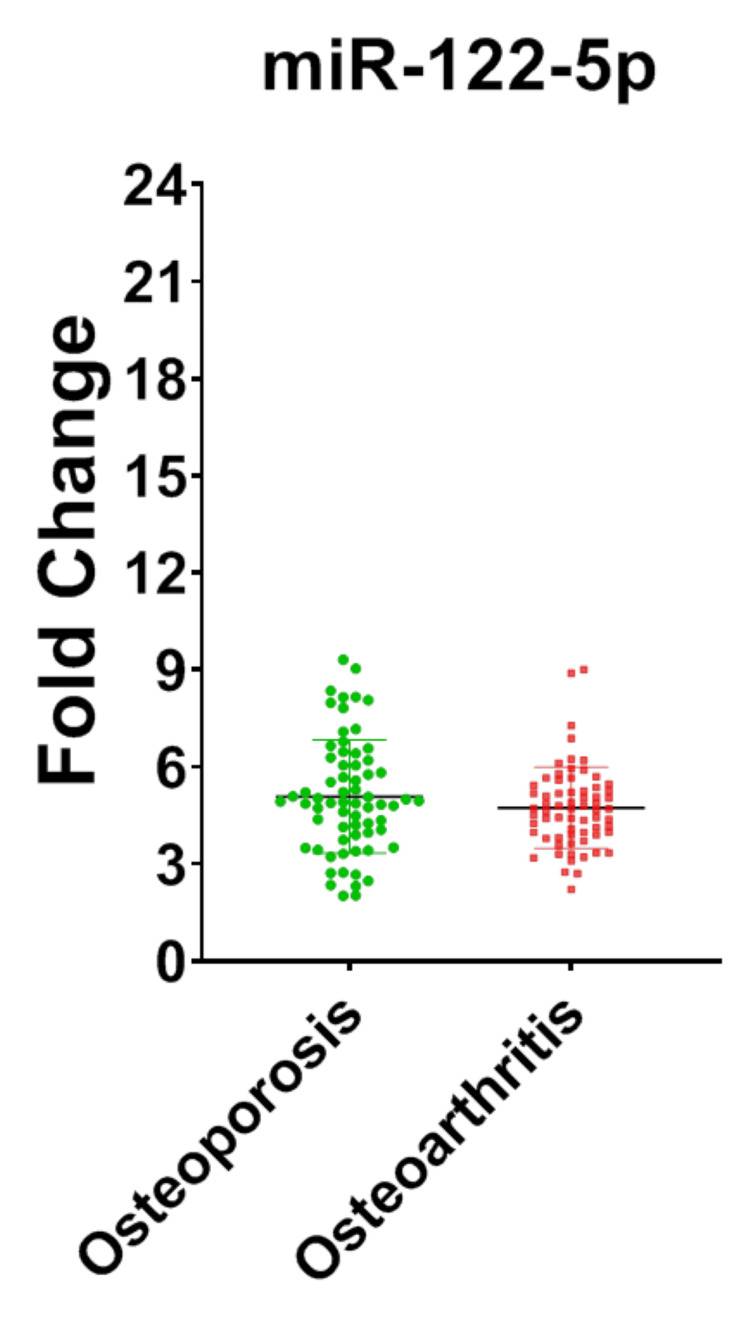
Dot plots were used to illustrate the differential distribution of miRNA levels (mean±SD) for miR-122-5p in knee osteoarthritis cases and osteoporosis cases. Each point on the plot indicates the individual expression level; the upper line represents the mean of the group, while the lower line represents the group's standard deviation. miR-122-5p, miRNA-122-5p

In Table 1, mean±SD of the age and gender are shown. In Table 2, individually the mean±SD is shown for each group. In Table 3, while comparing each group of cases with control, the expression of miR-122-5p is significantly upregulated. In OA versus control, the p-value (<0.0001) is significantly upregulated. In the OP versus control group, the expression of miRNA is upregulated. In the study group comprising common subjects with KOA and OP, the fold change shows a significant p-value, exhibiting that the miRNA expression is upregulated. In the comparison of KOA and OP, there is no significance. Figures 1-3 show the miR-122-5p levels that are upregulated. Figure 4 shows the comparison between KOA and OP, and the fold change value is 0.1868, which describes it as an insignificant value.

**Table 1:** Age and gender distribution of cases and controls.

Characteristics	KOA (n=67)	OP (n=67)	KOA+OP (n=67)	Control (n=67)
Age ± SD	55.7±9.21	60.06±8.4	59.19±7.0	55.33±6.65
Female:male	56:11	63:04	56:11	41:26

**Table 2:** Fold change in expression of miRNA in different groups.

Group	Fold change (mean±SD)
Knee osteoarthritis	4.73±1.25
Osteoporosis	5.08±1.74
Knee osteoarthritis + osteoporosis	10.92±3.42
Control	1.10±0.15

**Table 3:** Comparison of fold change of miRNA in different groups.

Group	P-value
Knee osteoarthritis vs. control	<0.0001
Osteoporosis vs. control	<0.0001
Knee osteoarthritis + osteoporosis vs. control	<0.0001
Knee osteoarthritis vs. osteoporosis	0.1868

In Table 4, the diagnostic potential of serum miRNA is given. The sensitivity and specificity of miR-122-5p are significant with a p-value of <0.0001 in all three groups: KOA vs. control, OP vs. control, and KOA + OP vs. control.

**Table 4:** Diagnostic potential of serum miRNA.

Mark	Group	Cut-off value	AUC	P-value	Sensitivity (95% CI)	Specificity (95% CI)
Serum miR-122-5p	Knee osteoarthritis vs. control	>2.310	0.99	<0.0001	100.0 (90.11-100.0)	97.14 (85.47-99.85)
Serum miR-122-5p	Osteoporosis vs. control	>1.945	0.99	<0.0001	100.0 (90.11-100.0)	97.14 (85.47-99.85)
Serum miR-122-5p	Knee osteoarthritis + osteoporosis vs. control	>3.335	1.00	<0.0001	100.0 (90.11-100.0)	100.0 90.11-100.0

ROC curve analysis

**Figure 5:**
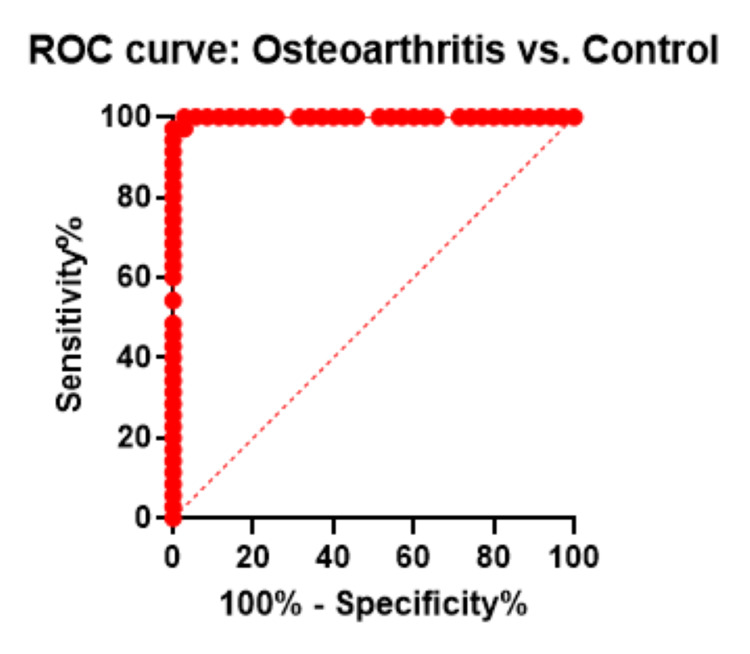
ROC curve for differentiation of knee osteoarthritis cases from controls. ROC, receiver operator characteristic curve

**Figure 6:**
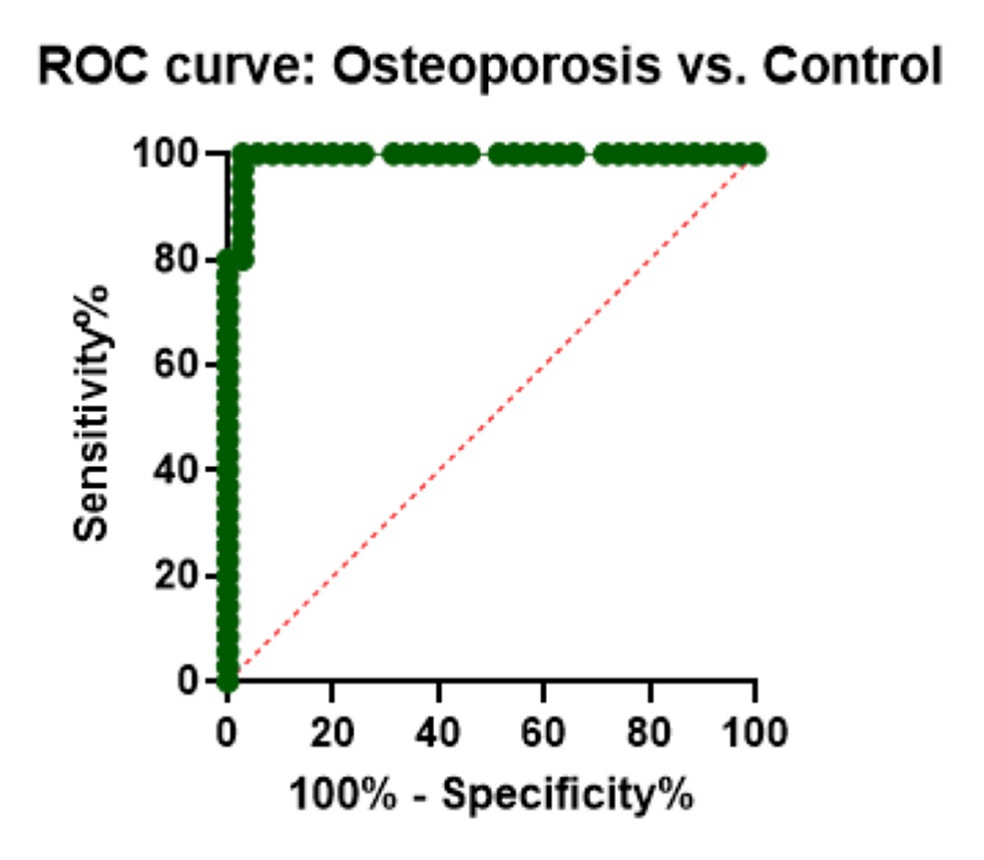
ROC curve for differentiation of osteoporosis cases from controls. ROC, receiver operator characteristic curve

**Figure 7:**
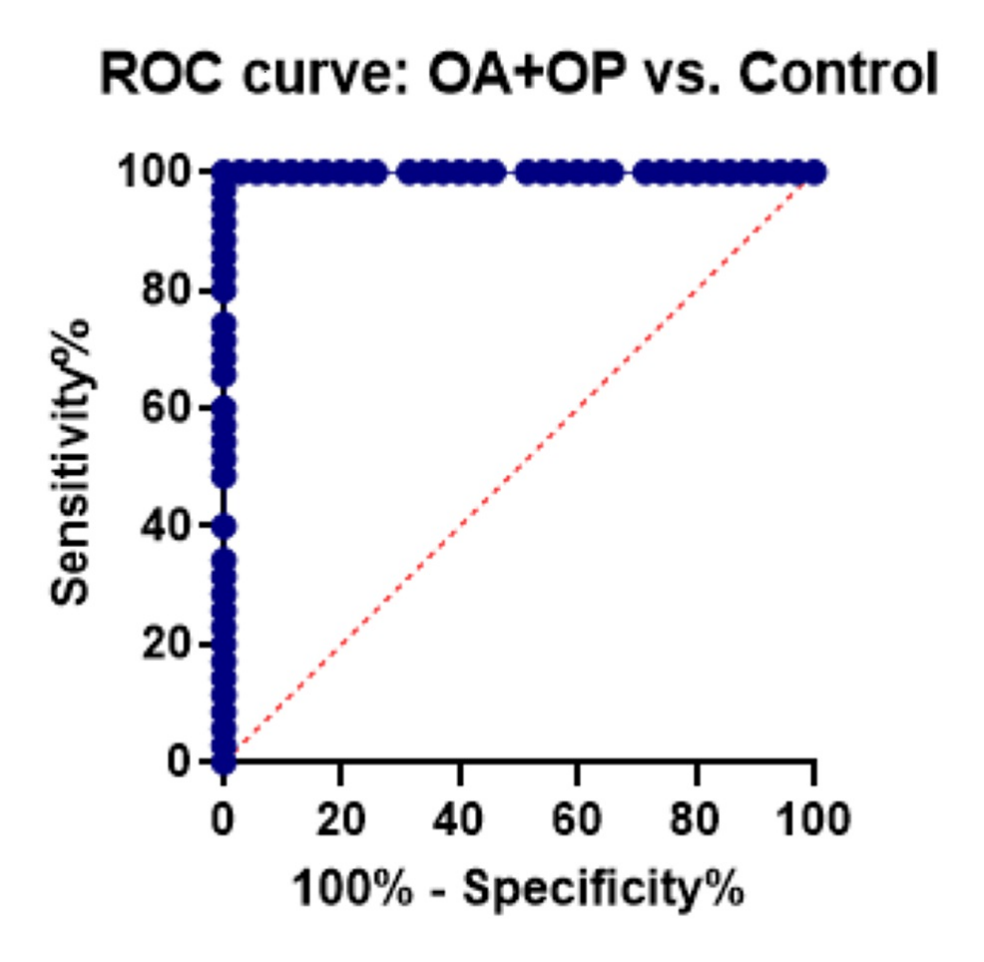
ROC curve for differentiation of osteoporosis with knee osteoarthritis cases from controls. ROC, receiver operator characteristic curve

A diagnostic method for analyzing the accuracy of the indicators could be used in relation to the ROC curve and the area under the curve (AUC). We calculated the ROC curve for each case versus control with the AUC value to evaluate the predictive value of upregulated miR-122-5p among cases. We obtained the following AUC values: 0.99 (p<0.000, 95% CI: 85.47-99.85) for KOA versus control, 0.99 (p<0.000, 95% CI: 85.47-99.85) for OP versus control, and 1.00 (p<0.000, 95% CI: 90.11-100.0) for subjects with both KOA and OP. Our results demonstrated that miR-122-5p has a great potential to provide sensitive and specific diagnostic value (Figures 5-7).

## Discussion

Recent findings indicate that circulating miRNAs can serve as noninvasive diagnostic and prognostic biomarkers for various diseases, including KOA, OP, and KOA and OP (subjects with both KOA and OP). In this study, we conducted a comparative analysis of circulating miR-122-5p levels in the serum of individuals with KOA, those with OP, those with both KOA and OP, and healthy controls using qPCR to assess their diagnostic value for individuals with both KOA and OP. Our results revealed elevated levels of circulating miR-122-5p in all three case groups as well as in healthy control subjects. Specifically, miR-122-5p levels were higher in individuals with KOA, OP, and those with both KOA and OP compared to healthy controls. Notably, the group with both KOA and OP exhibited higher miR-122-5p levels than the KOA and OP groups individually.

Several miRNAs play significant roles in KOA and OP independently, with miR-122-5p being one such crucial miRNA. Our study aligns with findings reported by Kong et al. [23], which identified upregulation of miR-19b-3p, miR-122-5p, and miR-486-5p as independent predictors of OA. Similarly, Kelch et al. [24] reported miR‐122‐5p as the most abundant miRNA in pooled samples of osteoporotic patients, showing a 15.79‐fold change compared to non-osteoporotic patients. Consistent results were observed in a study by Mandourah et al. [25] between osteoporotic and osteopenic subjects.

While some studies have reported a downregulation of miR-122-5p in clinical blood samples from OP versus non-OP subjects, others, including Zhang et al. [26], found a significant upregulation of miR-122-5p and miR-124-3p in serum and osteoclasts of osteoporotic patients. In a study, researchers observed that miR-122-5p expression was reduced in rabbits with osteonecrosis of the femoral head (ONFH). However, when miR-122-5p was overexpressed, it promoted osteoblast differentiation and osteogenesis. This effect was associated with increased expression of RUNX2 (a key transcription factor for bone development) and collagen I in osteoblasts [27]. In a clinical study, researchers discovered an association between miR-122-5p and miR-375 with bone deterioration. Specifically, miR-122-5p was upregulated in 15 postmenopausal women who had experienced a hip fracture [28]. In another study, the downregulation of miR-122-5p under the influence of indoleamine 2,3 dioxygenase 1 (IDO1) was found to be the crucial connection between IDO1 and the activation of the Wnt1/β-catenin signaling pathway in the stimulated mesenchymal stem cells [29]. These findings collectively suggest the potential utility of miR-122-5p as a diagnostic biomarker for these musculoskeletal disorders.

Furthermore, this study also supports the hypothesis that subjects having KOA (which occurs at an earlier age group) may be screened for the possibility of developing OP at a later age if this circulatory biomarker has levels. This, in turn, may facilitate more targeted and personalized therapeutic interventions, optimizing patient outcomes and improving the overall management of these complex musculoskeletal disorders. However, it is essential to acknowledge the limitations of our study, including its observational nature and the need for larger, longitudinal investigations to validate the utility of miR-122-5p as a biomarker.

## Conclusions

In conclusion, our study has provided valuable insights into the potential role of serum miR-122-5p expression as a circulatory biomarker in individuals affected by both KOA and OP. The coexistence of these two prevalent musculoskeletal conditions poses significant challenges in diagnosis, treatment, and management. Through our investigation, we aimed to shed light on the molecular underpinnings of this complex interplay and explore the feasibility of miR-122-5p as a diagnostic and prognostic tool. Our findings revealed a significant dysregulation in serum miR-122-5p expression in subjects with both KOA and OP compared to controls. The observed upregulation of miR-122-5p suggests its potential involvement in the intricate molecular pathways linking these two conditions. The association of miR-122-5p with processes related to inflammation, cartilage degradation, and bone remodelling provides a mechanistic basis for its candidacy as a circulatory biomarker.

The potential clinical implications of our findings are substantial. A reliable circulatory biomarker such as miR-122-5p could aid in early detection, risk stratification, and monitoring of patients having both KOA and OP. Our study provides the basis for future investigations that seek to understand the complex molecular landscape of musculoskeletal disorders and to facilitate towards the development of innovative therapeutic and diagnostic approaches.

## Figures and Tables

**Figure 8:**
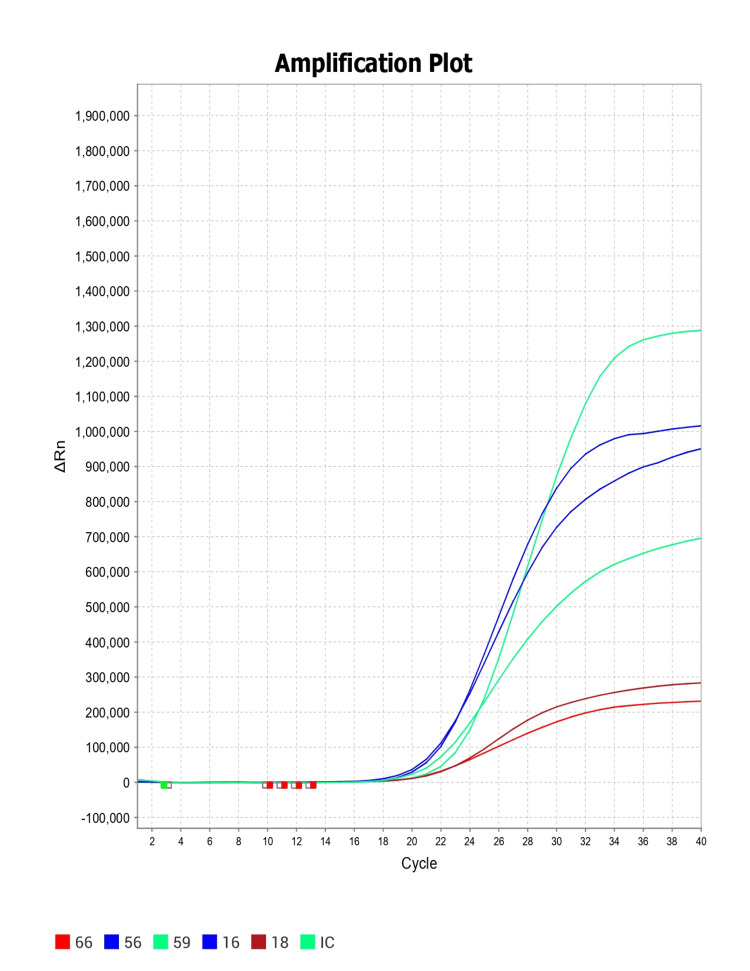
Amplification plot of miR-122-5p in cases having both knee osteoarthritis and osteoporosis. miR-122-5p, micro-RNA-122-5p
